# *Operando* studies of solid electrolyte interphase (SEI) formation with the electrochemical-surface force balance (e-SFB)

**DOI:** 10.1039/d6fd00040a

**Published:** 2026-04-08

**Authors:** Neave Taylor, Susan Perkin

**Affiliations:** a Physical and Theoretical Chemistry Laboratory, Department of Chemistry, University of Oxford Oxford OX1 3QZ UK susan.perkin@chem.ox.ac.uk

## Abstract

This study demonstrates the utility of the electrochemical-surface force balance (e-SFB) for observing solid electrolyte interphase (SEI) formation directly at an electrode surface. Through proof-of-concept measurements of lithium bis(trifluoromethanesulfonyl)imide (LiTFSI) based electrolytes, absolute thickness and mechanical properties of SEIs are measured *in situ* before and after electrochemical cycling. The SEIs that formed in these systems were found to have thickness on the order of 100s of nm and elastic moduli on the order of 10s of MPa depending on cycling conditions and the nature of the solvent.

## Abstract 2

This article demonstrates a new technique for measuring the thickness and mechanical properties of solid electrolyte interphases (SEIs) formed *in situ* on a gold electrode. Results are presented for two electrolytes.

## Graphical abstract

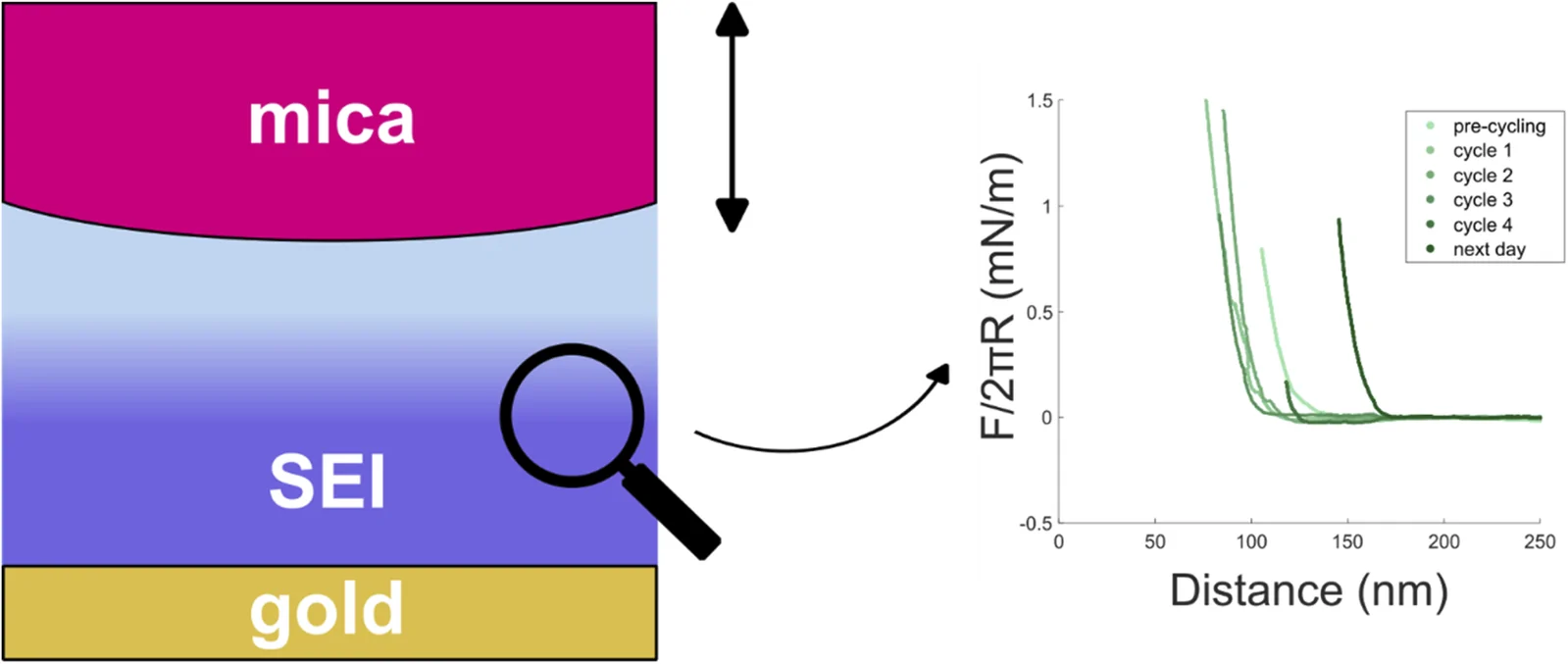

## Introduction

The surface force balance (SFB) allows us to measure the free energy of interaction between two surfaces across a liquid over a range of distances using white light interferometry. From interpreting these measurements, we can glean information about the nanostructure of a wide variety of liquids with sub-nanometre resolution. The SFB has previously been used to characterise some battery electrolytes, including those known to form an SEI,^1,2^ however studies to date have primarily used electrically insulating mica sheets as the measurement substrate. This limits the insights available into interfacial structuring at the range of potentials that an electrolyte would experience in a cycling battery. One of the mica surfaces can be replaced with a smooth, conductive gold surface whose potential can be readily controlled with a potentiostat.^3^

Efforts have already been made to study the morphology and composition of SEIs. However, comparatively little work has been done to measure and optimise the mechanical properties and behaviour of SEIs. The mechanical failure of the SEI during cycling is a major reason for battery failure, and so a better understanding of such properties is needed to prevent these modes of failure and improve battery performance and lifetime. The existing literature of mechanical studies on SEIs has recently been well summarised,^4,5^ and has made use of a diverse range of techniques such as computational modelling and simulations, atomic force microscopy (AFM), electrochemical quartz crystal microbalance (EQCM), and transmission electron microscopy (TEM). However, controlling the conditions of SEI growth to mirror the true conditions in a battery cell is difficult. Many measurements of the elastic moduli of SEIs have been conducted in dry, *ex situ* conditions where the SEI is known to be modified and damaged. Some measurements do paint a consistent picture, for example studies on lithium anodes have found a positive correlation between a higher SEI modulus and better battery performance.^6–8^ However, further precise *in situ* and *operando* measurements are needed to build a complete understanding of the mechanical properties. In addition, changes in the mechanical properties of the anode with cycling can be difficult to deconvolute from the SEI in many measurement geometries. Further, a recent review^4^ emphasised that the lack of direct measurements of SEI thickness (a quantity which should be readily evaluated using the e-SFB) is especially limiting to progressing understanding. Even though recent careful *in situ* AFM mechanical studies have been conducted on some electrolytes,^9–17^ questions still remain. In these measurements, SEI thickness is determined by inferring the contact point in AFM force curves, either by assuming the point where the AFM tip can no longer push through the deposited film is contact with the anode or by using scrape tests and finding the difference in distance between the scraped and unscraped areas. This likely leads to some systematic underestimation of the true SEI thickness as insufficient force may be applied to fully rupture the SEI or remove all of the SEI upon scraping.^18^

Here we demonstrate how the e-SFB technique can be applied to study the structure of the electrolyte, and any resulting SEI, at a variety of potentials directly measured at the interface with a gold electrode. This presents an opportunity to observe SEI growth and dissolution *in situ* and during electrochemical cycling. Compared to previous AFM measurements of SEI growth on potential controlled surfaces,^10,19,20^ the e-SFB has the additional benefit of absolute distances (rather than relative distances) being easily accessible, and by using an insulating probing surface (mica), lithiation of the measuring tip (possible when using a conductive AFM tip) is eliminated. Interactions are also averaged over a larger contact area than those seen by AFM tips or colloidal probes, which is useful for measuring quantities relevant to the bulk of the whole film and provides a gentler contact less likely to rupture the SEI. Using a gold thin film as the working electrode also avoids the complication of deconvoluting changes in the anode mechanical properties from the SEI throughout the measurements. To our knowledge, only one study has been published that uses the e-SFB to investigate SEI formation,^21^ but we believe this technique can deliver insights for a broad range of electrolytes of interest to the battery community.

In this *Faraday Discussions* article we present a control measurement in the e-SFB set-up of an ionic liquid within its electrochemical stability window. Then, measurements monitoring the growth and change of an SEI in LiTFSI WiSE (19 m) and LiTFSI in PC (1.8 M) are described.

## Methods

### Materials preparation

The ionic liquid 1-ethyl-3-methylimidazolium bis(trifluoromethylsulfonyl)imide ([C_2_C_1_Im][TFSI], IoLiTec, 99.5%) was dried on a Schlenk line at 10^−1^ mbar overnight at 30 °C. Lithium-salt solutions were prepared in a laminar flow hood (LFH) immediately before each experiment, with LiTFSI (Merck, 99.99%) weighed out first and then propylene carbonate (Merck, anhydrous 99.7%) or water (MilliQ IQ 7003, total organic content <5 ppb) added to make up solutions of about 5 mL in each case. The water content of the ionic liquid and propylene carbonate solution were measured immediately before and after each SFB experiment using the Karl Fischer Coulometric Titrator H I934. In the case of the ionic liquid and propylene carbonate measurements a small quantity of phosphorus pentoxide (∼1 g) was kept in a pot in the chamber throughout the course of the experiment as a desiccant. The refractive index of solutions was measured using the Abbe 60 refractometer with a sodium lamp.

### e-SFB measurements

**Fig. 1:**
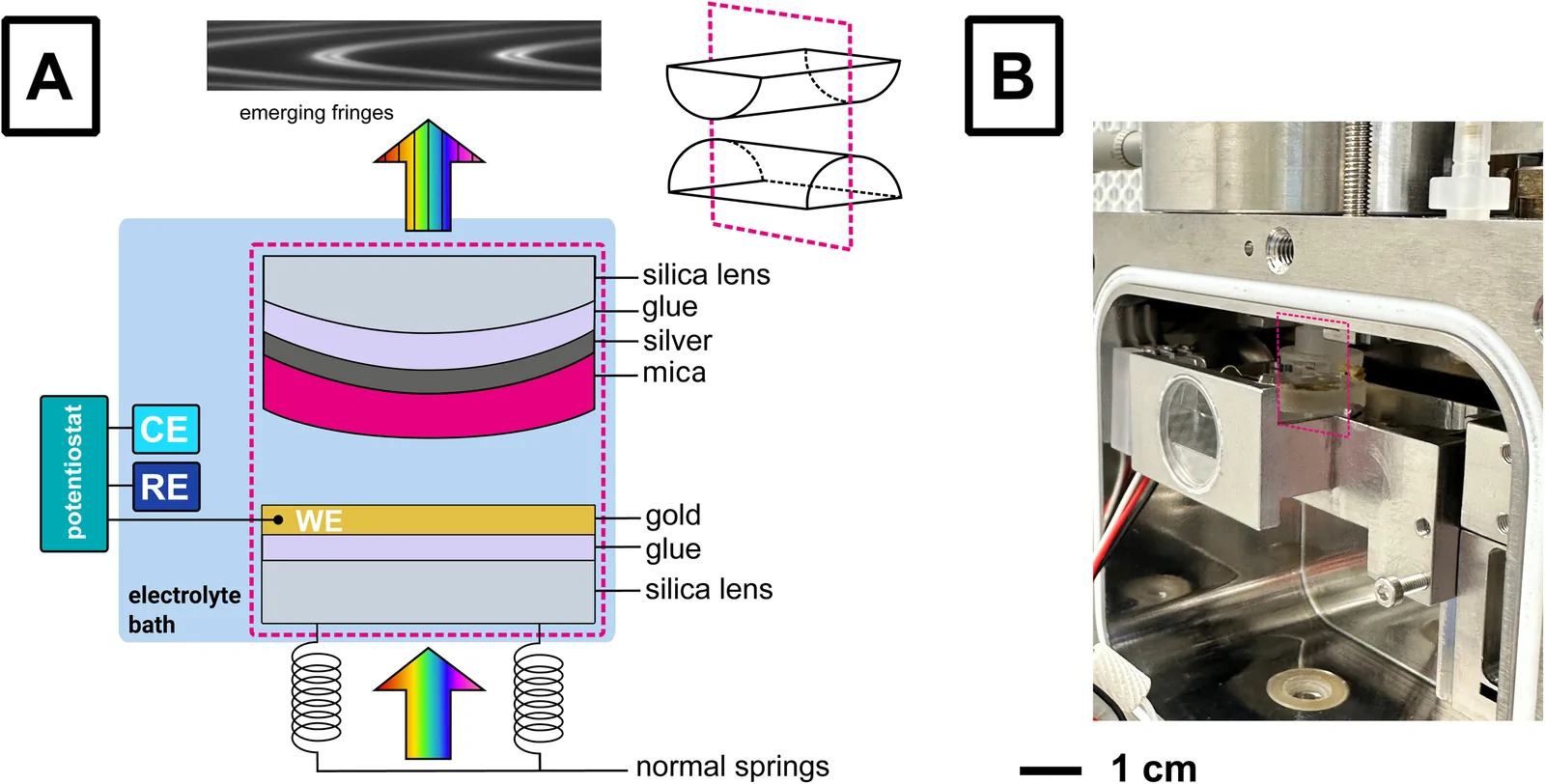
(A) Schematic illustration of the e-SFB set-up. (B) Photograph of lenses mounted perpendicular to each other in the SFB chamber.

The set-up is summarised in Fig. 1a. Ruby muscovite mica (S&J Trading Inc.) was cleaved in a laminar flow hood and gold was evaporated onto the clean face of the mica to a thickness of 45 nm. The gold-coated side of the mica was then stuck down onto a hemicylindrical silica lens (radius ∼10 mm) with epoxy resin (EPON 1004, Shell Chemicals) or glucose glue. Once securely glued, the mica was removed immediately before each SFB experiment to reveal a smooth and uniform gold film. This gold lens was then mounted on a horizontal leaf spring. Separately, silver was evaporated onto the back of another set of freshly cleaved mica pieces. Small pieces of mica (area ∼0.5 cm^2^) were cut with a scalpel and placed silver side down using epoxy resin or glucose on a silica lens. These lenses were then mounted opposite and perpendicular to each other in a cross-cylinder geometry in the e-SFB chamber as shown in Fig. 1b. A gold clamp connected to a gold wire was used to create a conductive path between the gold lens and the potentiostat (FRA 2 µAutolab Type III), allowing the gold surface to act as a working electrode (WE). Thin (approximately 1 mm diameter) wires were immersed in the electrolyte bath away from the lenses and acted as the (pseudo-)reference and counter electrodes. These wires were cleaned by sonicating in ethanol and soaking overnight in nitric acid (∼10%) before thorough rinsing with ultrapure water. In each experiment reported here Pt wire is used as the counter electrode (CE). Pt was used as the reference electrode (RE) in each experiment detailed here, except in the case of the ionic liquid where an Ag wire was used instead.

White light was shone through the lenses throughout the course of each experiment. The gold and silver surfaces are partly reflective and partly transmissive, resulting in an interference pattern which is a series of fringes of equal chromatic order (FECO). Images of these FECO with timestamps were the raw data recorded from which the distance between the two mirrors was precisely determined (to within ∼0.1 nm). The gold and mica surfaces were first brought into direct contact in air so that the mica thickness could be precisely determined. Enough of the liquid of interest was then injected to form a capillary bridge between the surfaces and immerse the ends of the reference and counter electrodes. A mechanical or piezoelectric motor was used to bring the surfaces together and apart. The velocity of translation of the surfaces towards each other was kept small (<1 nm s^−1^ for all data presented here) while the distance between the mica and gold surfaces for each frame of the measurement run was extracted from the raw data using analysis code previously developed using the principles of fast spectral correlation.^22^ The force was calculated using Hooke's law by extrapolating the expected linear displacement that would exist if there were no interaction between the surfaces from large distances down to small distances.

The potentiostat was used to conduct cyclic voltammetry (CV) experiments (relevant parameters included in the results section below) and/or hold the gold at a specified potential. A lack of applied potential is referred to as open circuit potential (OCP).

### Extracting mechanical information from e-SFB profiles

In order to predict how a material will behave under an applied force, its mechanical properties must be known. These can be determined from measuring the strain (*i.e.* deformation from the initial state) experienced by a material with a changing applied stress (*i.e.* pressure). Materials can deform elastically (reversibly) or plastically (irreversibly), may have a combination of behaviours or can be pushed to mechanical failure (*i.e.* fracture). One of the key mechanical properties of interest in the SEI community is the Young's modulus, the compressive stiffness of a material along the axis of the applied force. In the case of macroscopic materials, the Young's modulus is easily defined and routinely measured in industry. However, for micro- and nanoscale materials (the expected length scale relevant to SEIs) measurements become more subtle. For example, the elastic modulus and nature of attachment of the material to the measurement substrate can substantially change the apparent mechanical properties.^23^

To extract mechanical information from force–distance profiles, an appropriate contact model must be chosen. In the case of non-adhesive elastic contact, the Hertzian contact model is usually used to calculate the Young's modulus of materials in analogous AFM nanoindentation experiments^13,24^ where it is most appropriate at small indentation depths. In the geometry of a sphere-approaching a plane (equivalent to the crossed-cylinder geometry in the e-SFB), the force (*F*) is related to the indentation depth (*δ*) by:1
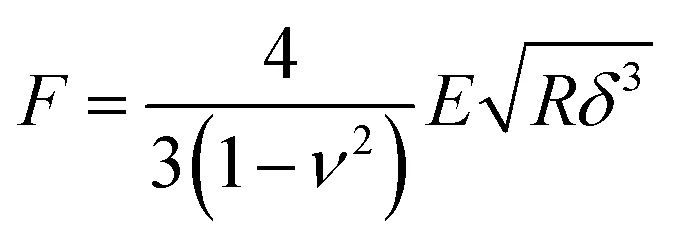
where *R* is the radius of the curvature of the lens, *ν* is Poisson's ratio for the material and *E* is the Young's modulus of the indented material. The Poisson's ratio captures the ratio of the transverse strain to the longitudinal strain in a material when it experiences compression or extension. It is challenging to measure directly and is not known for SEIs. In previous AFM studies it is assumed to have a value of 0.3–0.5.^13,24^ In our case a value of 0.3, typical for most solids,^25^ is assumed. We note that in the case of the LiTFSI WiSE film there is a plastic component to the structure (described in detail in the Results and discussion) which complicates this calculation. Nonetheless, in both the WiSE and PC systems the Hertzian model was used to fit for small indentations of the film to give an estimate of the bulk Young's modulus as a starting point.

## Results and discussion

### Control measurement

**Fig. 2:**
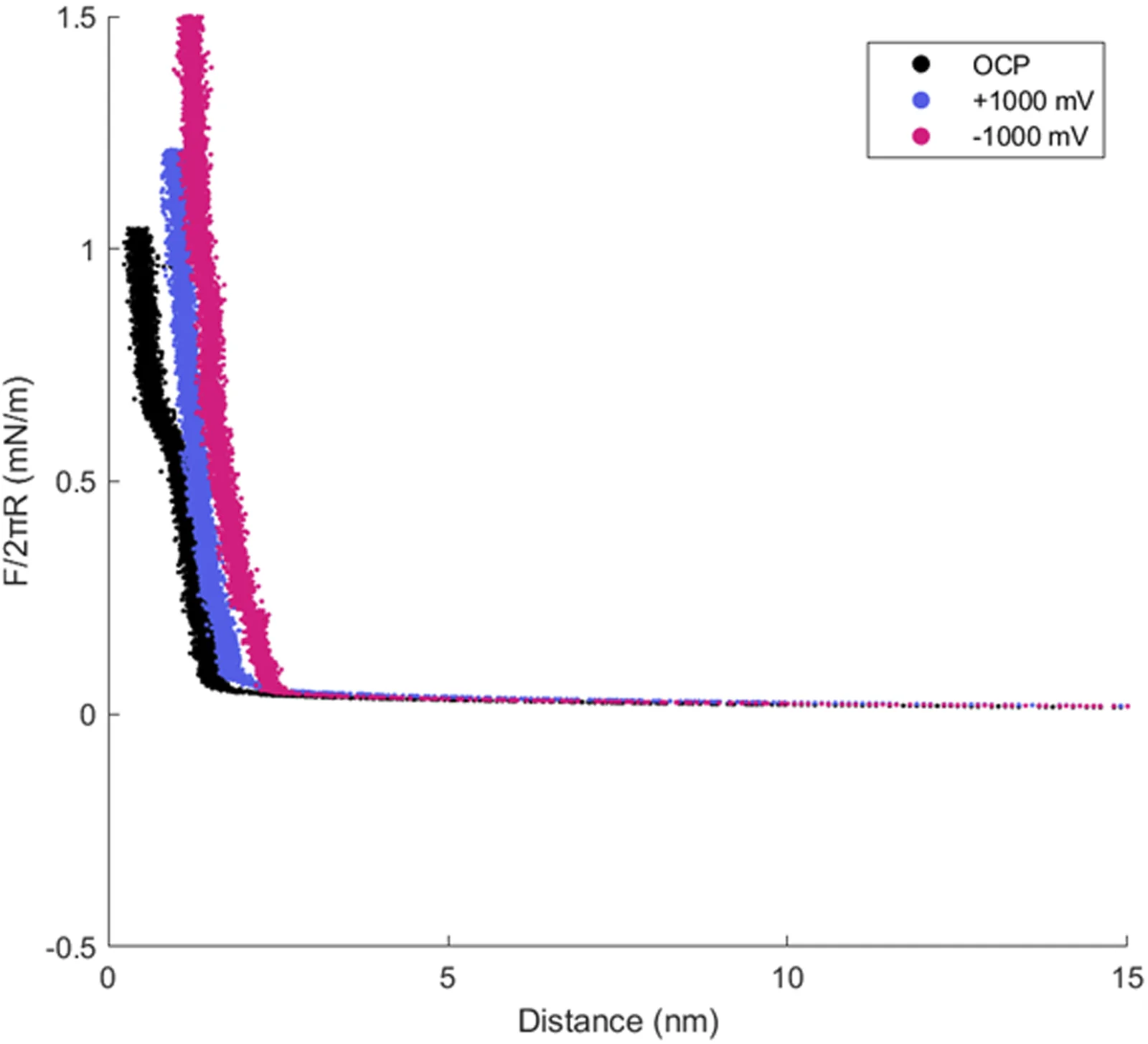
e-SFB surface forces approach measurements of [C_2_C_1_Im][TFSI] at OCP (black), +1000 mV (blue), and −1000 mV (pink) as measured against a Ag pseudo-reference electrode.

Before monitoring SEI formation, it was important to calibrate the set-up in a liquid that is not expected to form an SEI. The ionic liquid [C_2_C_1_Im][TFSI] was chosen because it has been well studied in the mica–mica SFB.^26^ This ionic liquid is known to have a wide electrochemical stability window even when wet,^27^ so we do not expect any electrochemical reactions to occur over a wide range of set potentials of the gold. This allows us to evaluate whether we have control over the potential of the gold consistently. Measurements of [C_2_C_1_Im][TFSI] in the e-SFB at various potentials within the electrochemical stability window are show in Fig. 2. No force is seen until the gold and mica surfaces are separated by about 3 nm, corresponding to only a few ion pairs. We could achieve close to complete contact between the surfaces, which suggests there were no surface contaminants on the gold surface in our preparation. Further, there is no evidence of any electrochemical decomposition products at any of the potentials studied.

### LiTFSI water-in-salt

**Fig. 3:**
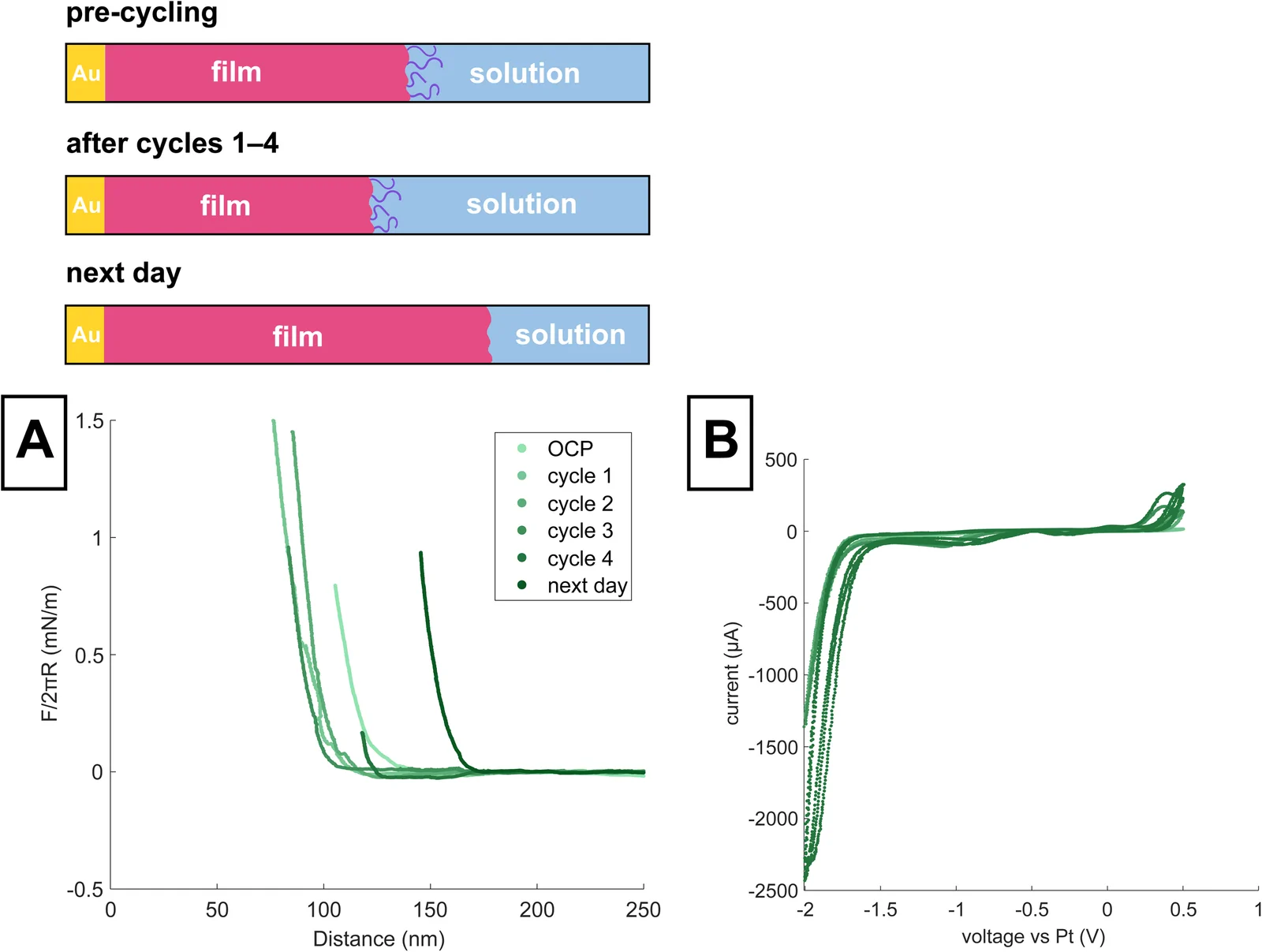
(A) Second approach profiles in LiTFSI WiSE (19 m) for before (OCP) and after one, two, three, and four cycles as well as after leaving the electrode in solution overnight (next day). A cartoon depicts a possible structure for the SEI. (B) Cyclic voltammograms as measured for each cycle.

The LiTFSI WiSE was prepared on the day of the experiment. Once the e-SFB was connected to the potentiostat 75 minutes after injection, an OCP of −53 mV (*vs.* Pt) was measured. The OCP decreased until stabilising at −81 mV (*vs.* Pt) over the course of about 75 minutes. This is the first point at which a force measurement was made. Representative force profiles obtained after running a galvanostatic cyclic voltammogram (CV) from the OCP to −2 V then to +0.5 V (*vs.* Pt) at 10 mV s^−1^ are shown in Fig. 3b. The large reductive peak is consistent with hydrogen evolution, found in previous work to be necessary for the formation of an SEI in this electrolyte.^28^ Approaching at OCP revealed a film with a thickness of ∼140 nm. As mica–mica measurements of LiTFSI water-in-salt electrolytes have not demonstrated such a film,^1^ we determine this must be a result of reactions between the electrode and electrolyte without an applied potential. The existence of a film pre-cycling is consistent with AFM work on other lithium-based electrolytes.^29^

**Fig. 4:**
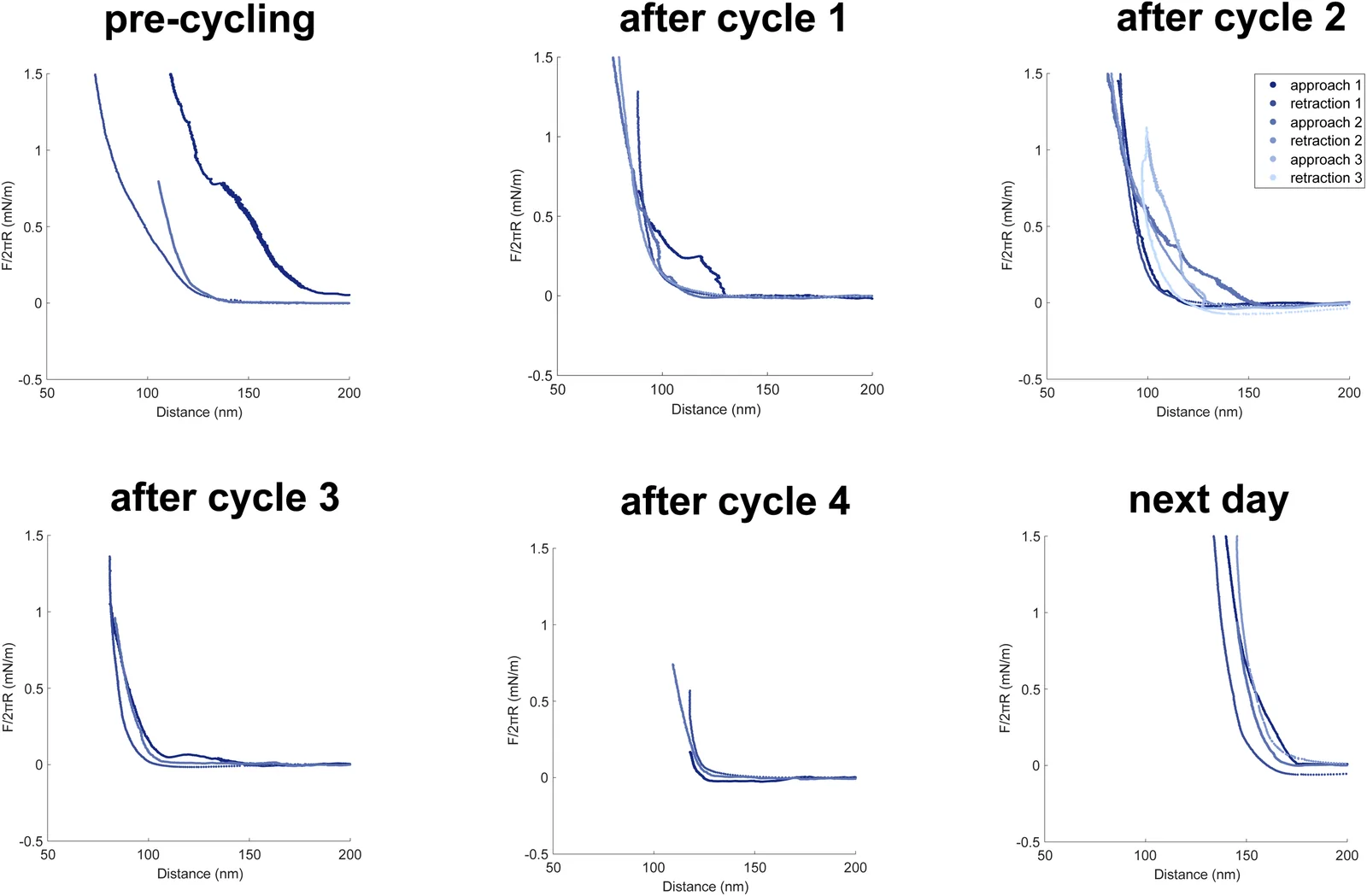
Overlay of approaches and retractions at different stages of cycling for LiTFSI WiSE.

After making multiple approaches at OCP to ensure consistency beyond the second approach, the lenses were taken far apart (>600 nm), and a CV was run. A comparison of the force profiles in sequence (first approaches, first retractions, second approaches and so on) for each point in cycling are shown in Fig. 4. For the pre-cycling case, as well as after cycles one and two, the first approach is markedly different to subsequent approaches and retractions. In each case the initial load seems to cause an irreversible compression of a region ∼20–50 nm thick which is not observed on the second approach. This would be best explained by the irreversible compression of a soft, dynamic outer layer. The compressed film after cycling is overall somewhat thinner (∼100 nm) than the uncycled film (∼140 nm). We propose that electrically cycling the electrode leads to some dissolution of the film, with the outer layer (*i.e.* facing the electrolyte) remaining dynamic throughout. The dynamic nature of the SEI has been reported elsewhere,^28,30^ although in the work by Jommongkol *et al.*^30^ the white film they observe could no longer be detected using *operando* optical microscopy when left at OCP for long periods. In contrast, the film we observe after leaving the set-up overnight grew thicker (up to ∼180 nm) and no longer contained an irreversibly compressible component. The partial dissolution of the film and the formation of a soft outer layer brought about by cycling of the electrode appears to be needed for the formation of the final thick, stable film.

**Fig. 5:**
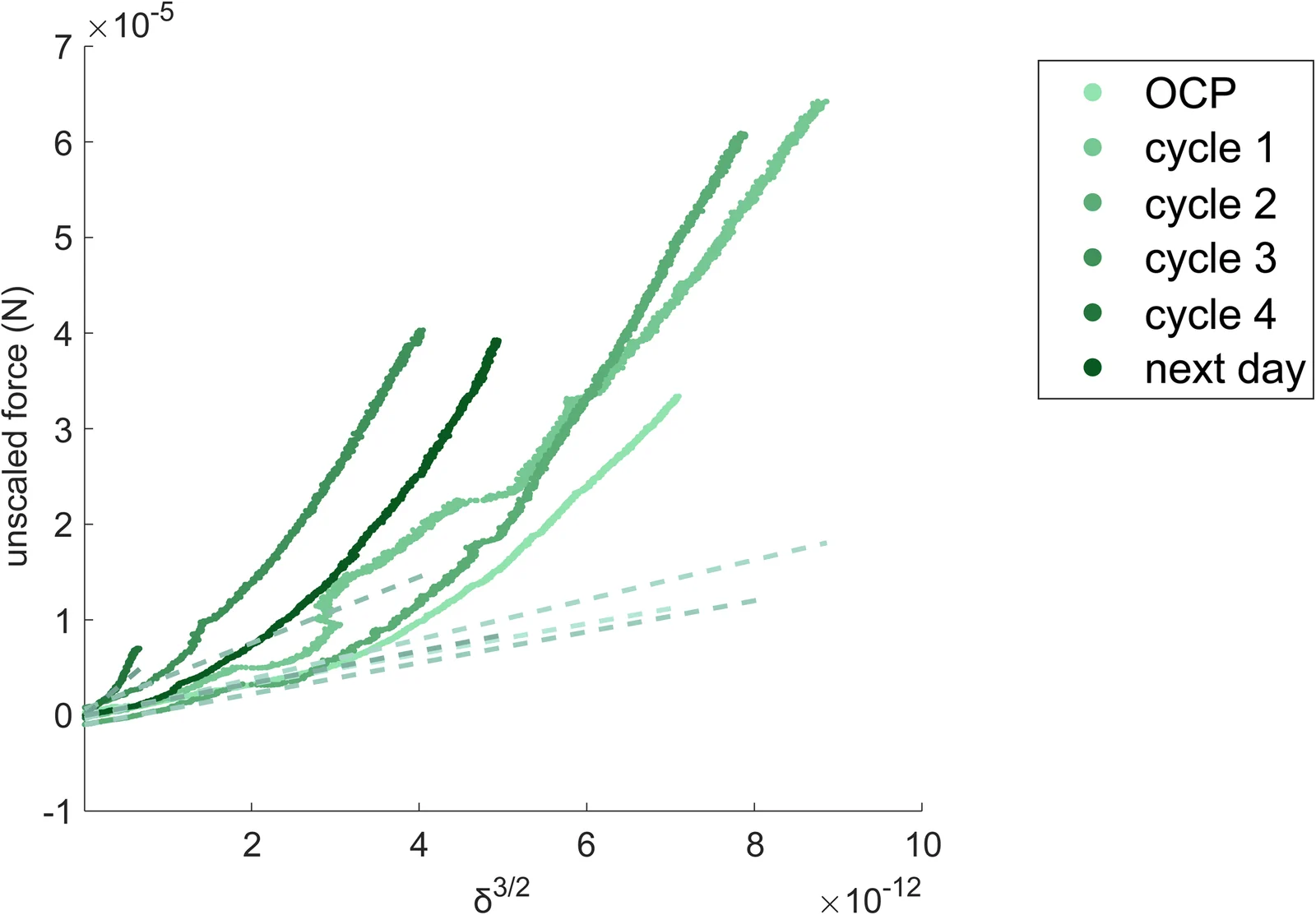
Force *vs. δ*^3/2^ for LiTFSI WiSE electrolyte second approach force profiles. Linear fits are shown as dashed lines.

From visual inspection of the second approach force profiles, we also determined the thickness of the elastic component of the SEI (*i.e.* the distance where *F* > 0 in these profiles). This allowed for a force *versus δ*^3/2^ graph to be drawn (Fig. 5). As the Hertzian model is only expected to be a reasonable fit for small displacements,^23^ we fit to the linear region at low *δ* and, using eqn (1), Young's moduli were calculated. At all stages of cycling the moduli were found to be in the range of 10–50 MPa without a clear trend with cycle number. This is consistent with the range of Young's moduli measured in other lithium-based electrolytes with electrochemical AFM *in situ* measurements.^14,31^

To our knowledge, only one other study of the thickness and mechanical properties of the LiTFSI water-in-salt electrolyte has been made.^11^ In the work by H. Zhang *et al.*,^11^ the thickness and Young's modulus of the SEI were measured *ex situ* after washing with dimethyl carbonate. A thickness of 4–6 nm and Young's modulus of 30 ± 10 GPa in some areas and 1–2 GPa in others was determined using AFM measurements. The discrepancy with our *in situ* results, where we find an SEI thickness of ∼100–140 nm and Young's moduli of 10–50 MPa, demonstrates that *ex situ* treatment of the SEI surface has a huge impact on the structure; removal from the original electrolyte and washing appears to lead to the loss of the majority of a softer, more extended SEI. These e-SFB results confirm that typical *ex situ* mechanical measurement protocols lead to very different conclusions on the thickness and mechanical properties of SEIs. It is possible that such measurements underestimate the thickness and overestimate the Young's modulus (due to only the stronger components of an SEI being likely to survive surface treatment) in other electrolyte systems as well, and so great care must be taken when using such measurements to guide better SEI design.

### LiTFSI in propylene carbonate

We begin discussion of the LiTFSI in PC (1.8 M) measurements by emphasising that these measurements could not be conducted in an air- and water-free environment. At the conclusion of the experiment, a high water content of >13 000 ppm was measured with a Karl-Fischer titrator despite the presence of drying agents and dry nitrogen gas flushing the measurement chamber. Much of this water uptake took place whilst preparing the electrolyte for measurement in dust-free but air-exposed LFHs.

**Fig. 6:**
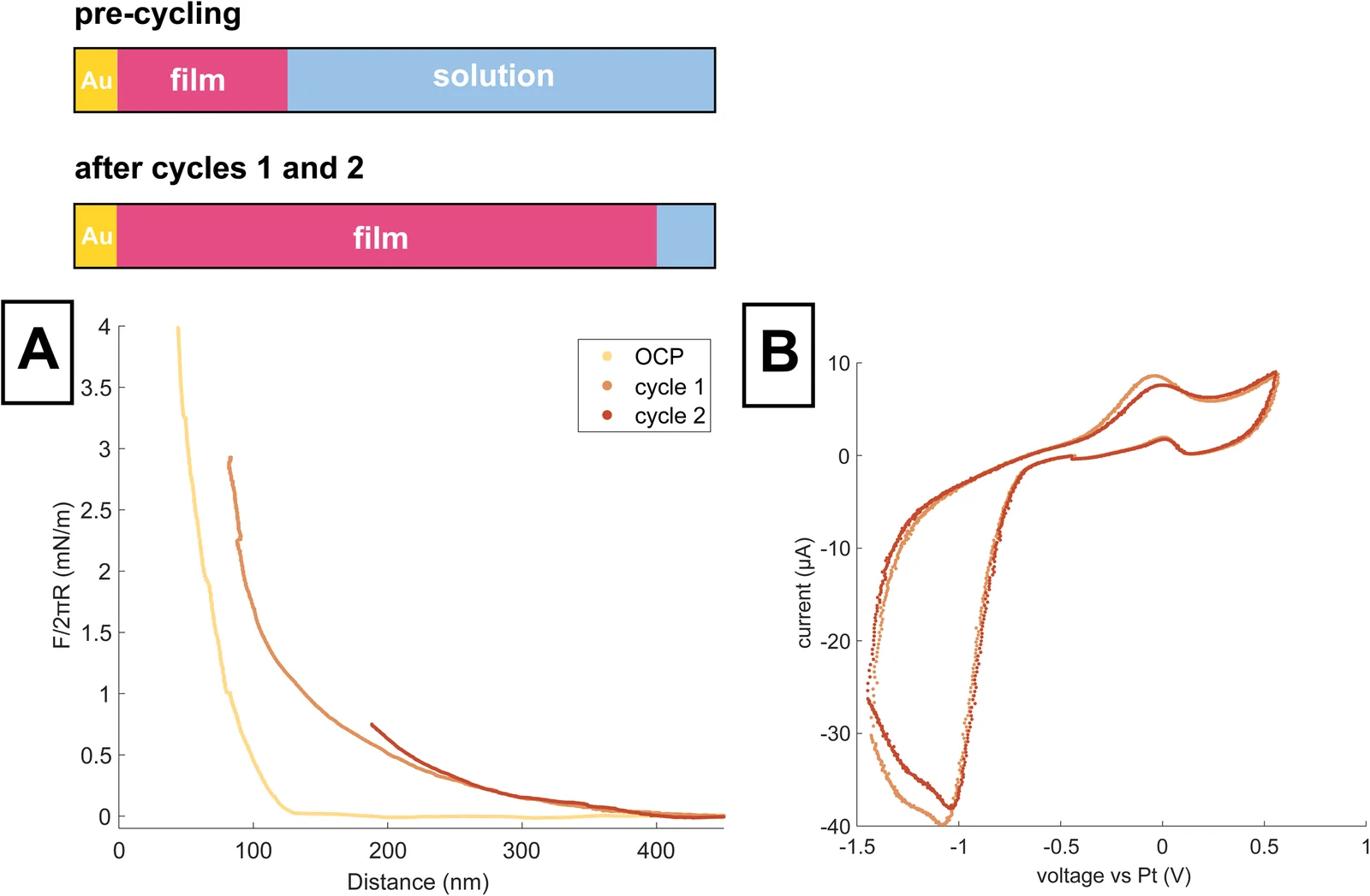
(A) Approaches in LiTFSI-PC (1.8 M) before cycling (OCP), after one cycle, and after two cycles. (B) Cyclic voltammograms as measured for each cycle.

**Fig. 7:**
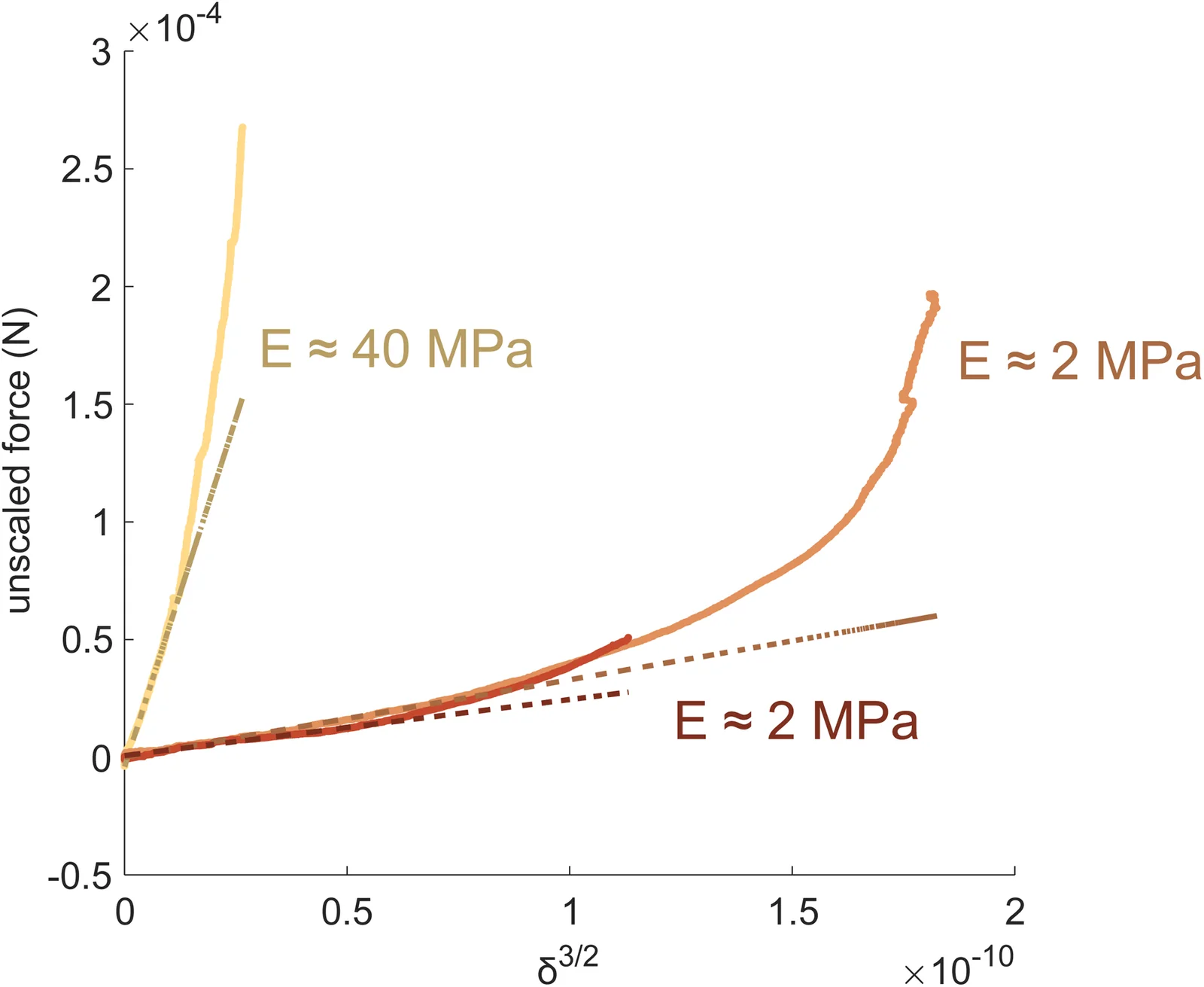
Force *versus δ*^3/2^ for LiTFSI-PC approach force profiles.

Representative approach force profiles are shown in Fig. 6a from before cycling, after one cycle, and after two cycles. Fig. 6b show the cyclic voltammetry run for each cycle at 10 mV s^−1^. The same fitting procedure to determine Young's modulus as described above were used to produce Fig. 7. A stiff (*E* ≈ 40 MPa) film with thickness ∼130 nm was observed before cycling. Much like in the WiSE case, this is consistent with a reaction between the electrode and the electrolyte without any potential applied to the gold. After one cycle, the film more than trebled in thickness (∼400 nm) but became much softer (*E* ≈ 2 MPa). There didn't appear to be any dynamic component of the structure irreversibly compressed in these measurements, so that is a feature specific to the WiSE case. After the second cycle, the film changed much less dramatically, becoming slightly thicker (∼420 nm) and remaining similarly soft (*E* ≈ 2 MPa). Based on the cyclic voltammograms (Fig. 6b), hydrogen evolution is strongly suspected to have occurred during these cycles. No further measurements were made in this experiment, as pushing the electrochemical window of the CVs wider after these two cycles resulted in delamination of the gold from the silica lens.

A few other studies have been made measuring the thickness and mechanical properties of LiTFSI in PC.^12,32^ P. C. Shi *et al.* found that the SEI that forms on graphite with the LiTFSI in PC electrolyte at a very similar concentration to the sample we studied (1.3 M) was uneven and had a thickness of ∼7 nm based on transmission electron microscopy images (TEM) after soaking and rinsing with ethyl methyl carbonate.^32^ In contrast, W.-W. Wang *et al.* examined LiTFSI in PC at a lower concentration (0.5 M) with and without a LiI additive. In the absence of the additive, the SEI was found to have a thickness of 80 nm with a modulus of 1.73 GPa from *in situ* AFM studies.^12^ The results from *in situ* measurements are closer to those found here (where the resulting films have a thickness of 100s of nanometres and Young's moduli of 2–40 MPa) than in the *ex situ* TEM measurement. Discrepancies with our measurements of this system with W.-W. Wang *et al.*'s findings are likely due to the effect of water contamination and the difference in concentration of the salt, but there is potentially also an underestimation of the SEI thickness from the *in situ* AFM indentation measurements.

## Conclusions

In this manuscript we have presented compression measurements of SEIs grown *in situ* for two battery electrolytes. We found that an initial film formed pre-cycling and that its thickness and stiffness evolved with time and cycling. In all cases, the thicknesses of the films were on the order of 100s of nanometres with Young's moduli in the range 1–50 MPa.

After the present proof of concept study, questions emerge to motivate future work:

• How much would the resulting SEI from these electrolytes vary when formed on different metal electrodes?

• To what extent would changing the cycling protocol, for example by varying voltage limits and scan rates, affect the results?

• How can we better account for the combined plastic and elastic behaviour of the SEI in the WiSE case?

In future, comparing the films that result from specific additives, or by systematically varying the concentration of the salt, is of great interest. Through correlation with battery performance metrics, clearer design rules for SEIs and electrolytes may become possible.

## Author contributions

S. P. and N. T. conceived and planned the experiments. N. T. performed the experiments and fitting. N. T. interpreted the data and wrote the paper with guidance and editing by S. P.

## Conflicts of interest

There are no conflicts to declare.

## Data Availability

All data for this article are available at the Oxford University Research Archive at https://doi.org/10.5287/ora-z60j5bnkn.
